# Foreign waste ban and green development of Chinese manufacturing industry: Comprehensive evidence based on provincial data

**DOI:** 10.1016/j.heliyon.2024.e38589

**Published:** 2024-09-27

**Authors:** Yanting Li, Xiaozhe Li, Fayyaz Ahmad

**Affiliations:** School of Economics, Lanzhou University, Lanzhou, 730000, Gansu, China

**Keywords:** Foreign waste, Solid waste imports, Environmental regulation, GTFP

## Abstract

Since the reform and opening up, imported solid wastes have pushed China's economic growth while also bringing about serious environmental problems. In this context, China has introduced a ban on “foreign waste” for imported solid waste. Based on the provincial panel data from 2010 to 2020, this paper calculates the Green Total Factor Productivity (GTFP) of the manufacturing industry to measure the level of green development of the manufacturing sector, and evaluates the comprehensive impact of the “foreign waste” ban on the green development of the manufacturing industry by using the continuous Difference-in-Differences method. The study finds that the foreign waste ban greatly promotes the greening of China's manufacturing sector, mechanism analysis shows that foreign waste ban stimulates innovation by innovation effects and increases production cost by cost effects, and the policy effect has heterogeneity as it is more prominent in coastal areas, areas with a lower proportion or a higher level of green development. This result suggests that the promotion of domestic solid waste recycling should be accompanied by further guidance on efficient factor mobility, with different measures based on regional heterogeneity.

## 1 Introduction

The issue of economic growth has always been the core of economics, and how to use limited resources to create maximum output through certain methods has become the focus of economic research. Since 1980s, China has made amazing success across many industries mainly in the manufacturing sector, but the crude growth mode driven by investment and factors has brought about inefficient use of resources and environmental pollution while realizing growth [1], lowered the GTFP, and sowed hidden dangers for the sustainability of economic growth. As resources continue to be consumed and emissions continue to increase, China urgently needs to realize a cleaner production pattern and seek a new growth path that balances productivity and environmental issues in order to avoid the low-end manufacturing and environmental pollution problems described in the “middle-income trap” [2].

However, a series of serious environmental problems have also come, today we need to achieve sustainable development by seeking a dynamic balance between natural resources, urbanization and economic growth [3], green development has become one of the important issues of the present era, the 20th Party Congress report is the “modernisation of man and nature in harmony and coexistence” up to the “Chinese-style modernisation” connotation. As the state attaches greater importance to green development, balancing the relationship between economy and conservation have also emerged as a priority, an array of environmental regulatory policies have been introduced by the government to alleviate the contradiction [4]. Environmental regulation refers to the government's direct intervention in environmental resources by various means, forcing enterprises to consider their own externalities on sustainability in their production and business activities. Unlike the market-based environmental regulation of Western countries, China mainly adopts the environmental regulation of orders issued or assessed by public authorities [5]. The ban on “foreign waste” is one of them.

The ban on “foreign waste” is a restrictive policy on the import of solid waste promulgated by China in 2017. The so-called solid waste refers to solid materials generated in the course of production, consumption, living, and other activities, while “foreign waste” mainly refers to solid waste whose import is banned or restricted. In view of global circular economy, final products are used for consumption in developed countries while waste left is collected for recovery and utilization in developing countries [6,7]. This practice significantly reduces disposal costs for high-income countries, which are the main producers of waste, but imposes an enormous environmental burden on the importing countries concerned and on the whole international community.

For decades, China has been one of the world's leading destinations for the recycling, reuse and disposal of solid waste from all over the world, particularly from developed countries [8]. At the beginning of the reform and opening up, to solve the problem of shortage of raw materials and further develop productivity, China began to import large quantities of solid waste, but with the promulgation of the Basel Convention in 1989, the country also began to pay attention to this kind of “transfer of pollution” from developed countries gradually. In the 1990s, the State Council issued the Notice on Strictly Controlling the Transfer of Hazardous Wastes from Overseas Countries to China, the Emergency Notice on Resolutely Controlling the Transfer of Wastes from Overseas Countries to China, and other documents, and at the same time introduced a series of regulations and standards to start controlling the importation of hazardous wastes and waste. “In April 2006, the government issued the Guidelines for Identification of Solid Wastes, which further strengthened restriction, and in 2011, they issued the Guidelines for Imported Solid Wastes Useable as Raw Materials, while issuing another policy to further improve the management of the importation of certain solid waste, and the Customs initiated the “Green Fence” special action in 2013 to carry out a nationwide campaign against imported wastes. In 2013, Customs launched the “Green Hedges” special operation to carry out special rectification work on imported wastes nationwide. By implementing a series of policies and regulations, the State has begun to gradually improved the management of imported solid waste, in July 2017, the State Council formally launched the ban on “foreign waste”, which clearly stated that by the end of 2017, it would ban the import of unsorted waste paper and other solid wastes that are of great environmental hazards and have been strongly reported by the public from domestic sources, and formally imposed strict regulations on the import of solid waste. Thereafter, in November 2020, the State Council promulgated an Announcement, announcing that an outright prohibition on the import of solid wastes would be imposed from January 1, 2021 onwards [9].

Although imported waste has brought many benefits to China, melting down plastic waste for recycling can produce volatile organic compounds (VOCs) [10,11], and in the presence of sunlight, these VOCs react with NOx to create low-level ozone [12], Such contamination is damaging to public health and causes significant losses, especially in a booming market such as China [13]. The latest research points to effects of air pollution, for example the reduced performance on cognitive tasks [14], loss of labor productivity [15], and sleeplessness [16]. As a major waste importer, China is bound to receive a significant impact from the foreign waste ban, and based on the above discussion of the numerous effects of waste imports, this impact is worth studying.

The implementation of the “foreign waste” ban is an inevitable requirement for realizing sustainable development, but it has not yet been concluded whether this policy can really play its due effect and further realize the unity of economic efficiency and environmental governance. Existing researches on the foreign waste ban focus more on the international impact of this policy, with research topics clustered in areas such as the global circular economy [8], global waste trade [17,18], and the global recycling industry [19], and few scholars have investigated the effects that this policy has had on the greening of economy. Therefore, based on existing research, this paper will take Green Total Factor Productivity (GTFP) as the main indicator, explore the comprehensive effect of the “foreign waste” ban on China's green manufacturing development, the specific mechanisms and the heterogeneity of its role.

This paper has following contributions: Firstly, it examines whether and how foreign waste ban affects the greening of the Chinese manufacture sector. Foreign waste was initially used mainly for the growth of manufacturing industry, and the ban was originally introduced to ensure the green development of China. This paper empirically investigates the impact of the ban by estimating GTFP, which is a comprehensive indicator that integrates the environmental issues and the growth issues, and fills the gaps in the existing research. Secondly, it broadens the perspective of related research. Most of the existing environmental regulations are direct or indirect restrictions on emissions from the output side, while the ban on “foreign waste” is a direct control of raw materials from the input side, so the study of the policy effect of this ban is an important addition to the existing research on environmental regulation. Thirdly, the analysis of the specific effects of environmental regulation is further refined. By breaking down the specific effects of the “foreign waste” ban as cost effects, innovation effects and resource allocation effects, it reveals the specific paths of such environmental regulations on the manufacturing industry, and provides a possible basis for subsequent policy selection and formulation.

**Fig. 1:**
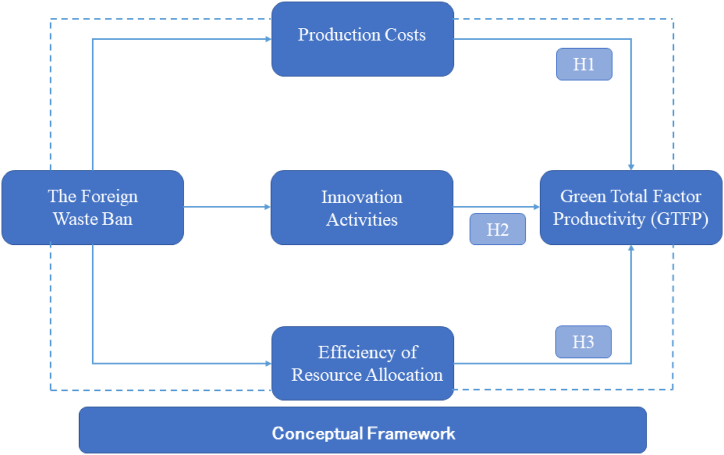
Conceptual framework.

Our conceptual framework is shown as Fig. 1. Based on this, we first conduct a summary of the existing research and proceed with the theoretical analysis accordingly, then, we explain the selected methodology and sample data sources, followed by data analysis and reporting of the results. Finally, we summarize and discuss the research and propose research implications based on the results of the study.

## 2 Literature review

Environmental regulation mainly refers to the government's direct intervention in environmental resources by non-market means, forcing enterprises to take into account their externalities on natural resources and the ecological environment in their activities, which affect both the economy and the environment. As a comprehensive indicator for rationally evaluating economic and environmental benefits, the change of GTFP can become a standard for assessing the actual effect. The urgency of environmental problems has led scholars to think about the connection which exists between environmental legislation and sustainable economic growth. Current research on the impact of environmental regulation on GTFP mainly focuses on three effects, namely the cost effects, innovation effects and resource allocation effects.

Regarding the cost effect, some studies have found that such policy will negatively affect GTFP. First of all, some seriously polluting enterprises will be forced to reduce production to cope with environmental regulations, thus reducing the GTFP [20], In addition, enterprises will spend more costs on technological progress, thus cut down investment in other areas in the formation of the “crowding out effect” [21], which may ultimately lead to the production, profitability of the manufacturing industry. This may ultimately lead to a reduction in production and profitability. In terms of the innovation effects, according to the viewpoint of neoclassical economics, the usage of such policies will counteract the positive effects of environmental protection on society by increasing the production costs of companies and reducing their competitive capacity, and generally playing a negative effect on economic growth. However, American economist Michael Porter challenged this claim in 1991, arguing that certain environmental protection policies not only do not increase the cost of enterprises, but also incentivize innovation, help their profitability, and thus improve the international competitive advantage of enterprises [22], which is the Porter's hypothesis. In addition to directly encouraging enterprises to save energy and reduce emissions, reduce environmental pollution, and improve GTFP, relevant studies have also tested regulation's positive impact on GTFP in manufacturing industry by verifying the existence of the “Porter effect”. Some studies believe that in the long run, green production and consumption under environmental regulation will form a new competitive advantage to offset the negative effects of rising costs: firstly, the government has a key role to play in encouraging businesses to innovate in green technologies, according to Porter's hypothesis [[23], [24], [25]], environmental regulation through the mediating variable “technological innovation”, compels companies to innovate independently, technology introduction and other activities, promotes the technological progress of enterprises, and improves the production efficiency of enterprises through technological improvement, which ultimately has a great impact on the GTFP [[26], [27], [28]]. Secondly, In order to meet government requirements, companies subject to environmental regulations often engage in green innovations to mitigate the emissions they produce, and studies have shown that in addition to directly mitigating pollution, green innovations can increase the returns of the stocks listed [29], alleviate energy scarcity in some countries by improving energy efficiency [30,31], it also has significant relationship with CO2 emissions and ecological footprint [[32], [33], [34], [35]], integrate green innovation and CO2 emissions can be mitigated significantly through balanced environmental policy enforcement and energy transition measures. [32], therefore green innovation has a strong effect on GTFP enhancement. Thirdly, the government's environmental regulation means to change the traditional competitive environment, this external stimulus can help enterprises to explore the potential market space or opportunity to form a first-mover advantage [36]. Therefore, scholars believe that the government's intervention in corporate behavior through reasonable environmental regulation can play a strong role in promoting green development, both from the perspective of enterprises and macroeconomics. In terms of resource allocation effects, research shows that resource allocation efficiency enhances GTFP [37,38], and such policy promotes resource allocation efficiency: on the one hand, environmental regulation will optimize the resource allocation efficiency among enterprises, improve the exit probability of inefficient enterprises [39], eliminate highly polluting enterprises with lower technology level, and realize the improvement of GTFP [20]. On the other hand, environmental regulation will promote the reallocation of resources within the enterprise and promote the upgrading of product structure [40].

As a form of environmental regulation, the ban on “foreign waste” could also play a role in the green development of the manufacturing sector through similar effects. The term “foreign waste” refers to imported solid wastes, and sometimes specifically refers to unauthorized solid wastes that are prohibited or restricted by the state through smuggling, entrainment and other means of entry, which is more often defined as the latter in various studies. As the largest country in the global waste trade, China's annual waste imports account for more than 50 % globally, which promotes economic growth but also brings about greater environmental pollution and greatly jeopardizes people's health [41].

The import of solid waste has brought about a series of impacts on China. Over the past four decades, imported solid waste has become the raw material for some enterprises in China, greatly reducing production costs and promoting the development of various industries, especially the manufacturing industry, in addition, Wang [42] thought that the sorting and recycling of “foreign waste” is a labor-intensive industry, which has brought job opportunities for poorer rural residents to improve their incomes. At the same time, “foreign waste” is a labor-intensive industry. At the same time, the import of “foreign waste” also brings a series of environmental problems, causing environmental pollution and endangering people's health. From the different characteristics of various types of “foreign waste”, they are bound to cause diversified pollution to the ecological environment. Wang et al. [43] found that the rough way of operation and the secondary pollution brought by processing and reuse further aggravate the degree of pollution, and the pollution brought by “foreign waste” also shows the characteristics of a wide range of pollution, strong harm, and a long recovery cycle. In addition, in recent years, China's “shortage” has become a major problem. In addition, in recent years, the improvement of China's “shortage economy” phenomenon has led to a decline in the urgent need for such raw materials, and the public opinion against the entry of “foreign waste” has become increasingly strong, so that the joint effect of environmental, economic and social factors has brought about the ban on the import of the inevitability of waste import [44], the ban on “foreign waste” was introduced.

“Foreign waste” and China's manufacturing production process have a strong correlation, and the effect of the ban on the manufacturing industry also has a strong reflection. The promulgation of the policy directly reduces the pollution caused by waste disposal, and in the short term forces manufacturing enterprises to improve the efficiency of resource utilization and technological innovation; and most of the enterprises importing “foreign waste” as raw materials belong to “scattered and chaotic polluted” enterprises, with poor ability to control pollution [45]. Therefore, in the long run, the ban promotes the improvement of the overall efficiency of the manufacture sector through the elimination of inefficient enterprises, promotes industrial transformation and upgrading of domestic solid waste recycling and processing and utilization, promotes the rectification and protection of the domestic ecological environment, and regulates the relevant industries, which plays a certain positive role [46]. However, at the same time, it also brings certain negative impacts: on the one hand, Li [47] found that it directly led to an increase in the cost of production of some enterprises, this impact is particularly reflected in the paper industry, where waste paper raw materials accounted for 65 % of China's paper and related industries raw materials, but due to the low rate of recycling of waste paper and recycling technology is underdeveloped, China's domestic recycling of waste paper can be less, therefore, papermaking and related industries need a large part of the waste paper raw materials Part of the raw materials for paper and related industries come from imports. On the other hand, the ban will have a short-term impact on the recycling industry, bringing the risk of closure to many related enterprises [45], thus indirectly affecting the raw material structure of manufacturing enterprises.

The current research on the ban of “foreign waste” is mainly divided into two aspects of international and domestic impacts: in terms of international effects, Xiong et al. [48] analysed the process of China's ban on importing “foreign waste” and elaborated on the impacts of the shift of solid waste export focus from developed countries to Southeast Asian countries in the short term, the substantial reduction of overall solid waste export in the medium term, and the gradual shift to self-production and self-sale in the long term, while Wang [49] also takes many countries as examples to illustrate that China's ban has caught the waste-exporting countries by surprise and begun to increase the capital investment in waste treatment; in terms of the domestic effect, Li [41] conducted a study through the life cycle of foreign waste trade process at all stages of carbon emissions caused by the evaluation, accounting and comparison, and waste paper imports and related paper industry as an example, predicted the “foreign waste” trade in different scenarios of carbon emissions and the average annual wastewater emissions of domestic paper enterprises, and further analyse the solid waste restrictions on imports of China's related industries. Zhang et al. [50] analysed the economic effect of the ban on China by taking the waste paper industry as an example. However, many studies are limited to the surface of the theoretical analysis, which is not sufficient, and the lack of a comprehensive evaluation of the policy effect, few scholars have assessed the impact of this solid waste import restriction policy through quantitative means, N and T [51] assessed the environmental impact of the ban on imports of Japanese plastics by calculating the change in CO_2_ emissions after the ban on imports of Japanese plastics.

However, such studies have several limitations, both in terms of the subjects studied and the methods of evaluation. They didn't consider the relationship between this policy and the development of the manufacture sector, which is the sector most affected, and they didn't take into account the comprehensive impact of this policy, which made their indicators restrictive. As a comprehensive indicator, the change of GTFP can be a standard to assess the effect of this policy, and the ban on the collection of “foreign waste” will inevitably produce various effects in terms of the economy and the environment, thus affecting the GTFP. The ban on “foreign waste” will inevitably produce various impacts from economic, environmental, and other aspects, thus affecting the GTFP. From the above analysis, we can see that the effect of the ban has a central embodiment in the manufacturing industry. Sustainable development is a worldwide issue, at the same time, from a theoretical point of view, as a kind of environmental regulation, exploring the ban on the greening of China's manufacturing sector can make a possible marginal contribution to the existing environmental regulation, and the current mainstream environmental regulation is mainly from the output side of the direct or indirect control of corporate emissions, and the ban directly from the input side of the raw materials to play a role, so it is capable of broaden the perspective of related research. Therefore, this paper constructs a double-difference model to assess the policy effect of the ban on “foreign waste” through the indicator of GTFP, so as to explore the comprehensive effect of the ban on the green development of the manufacturing industry under the multiple paths of action, to enrich the relevant research results, and to provide certain theoretical and practical significance.

In summary, the ban on “foreign waste” will undoubtedly bring some degree of positive and negative impacts on the economy and environment both at home and abroad, and this impact is mainly reflected in the manufacture sector in China, but the comprehensive effect of the policy on the manufacturing industry is not yet clear. Therefore, this paper selects the indicator of GTFP to explore the policy effect of the ban on “foreign waste”, aiming to study the shock of this policy on the manufacturing industry under the role of multiple factors, and further explore the role of its role in the heterogeneity and the mechanism, and ultimately put forward the corresponding optimization measures and policy recommendations on this basis.

## 3 Theoretical analysis

**Fig. 2:**
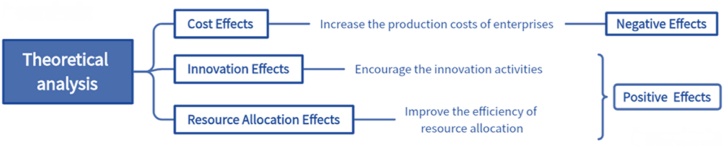
Theoretical analysis of different effects.

Fig. 2 shows the path of theoretical analysis, where the cost effect is shown as a negative impact, while the innovation effect and the resource allocation effect are shown as positive impacts.

### 3.1 Cost effects

With the implementation of the ban, the raw material structure and economic efficiency of manufacturing enterprises have been significantly affected. On the one hand, solid waste, as a cheaper raw materials, is often recycled by manufacturing enterprises to buy and put into production at low prices, thus reducing production costs, and due to technical constraints, the degree of development of waste classification and recycling limitations, imported solid wastes are often of higher quality, more popular with the manufacturing industry, so the ban on “foreign waste” Therefore, the implementation of the “foreign waste” ban directly reduces the supply of raw materials to enterprises, increasing their production costs. On the other hand, after the implementation of the ban, foreign waste is reduced, while the residents are not aware of waste classification, the effectiveness of waste classification is not good, the raw materials from domestic waste are not enough, the ban caused by the reduction of raw material sources of renewable resources recycling enterprises to the recycling industry impact, so for the manufacturing industry, a large number of low-end recycling enterprises will be faced with elimination of the fate of the closure of the manufacture sector, the industry from imported and domestic solid waste raw materials reduced and domestic solid waste will be reduced and costs will rise. Accordingly, the following assumptions are made.

H1 the ban on “foreign waste” would increase the cost of raw materials for manufacturing firms by changing their raw material mix, thereby reducing GTFP in manufacturing.

### 3.2 Innovation effects

For manufacturing firms involved in extensive import and export trade such as importing foreign waste, avenues like buyer-driven knowledge transfer activities [52] and supplier engagement [53] will undoubtedly contribute directly to innovation, while the foreign waste ban as an environmental regulation will also play an important role here. According to Porter's hypothesis, a certain degree of environmental regulation is likely to incentivize firms to increase R&D and technological innovation to improve production efficiency, which not only helps firms to meet needed standards, but also compensates for or outweighs the initial costs of the regulation. The ban on “foreign waste”, as a form of environmental regulation, is likely to have a similar effect: it will force affected firms to find new sources of materials, adopt new production processes or improve existing technologies. This will mean higher R&D expenditure and production pressure in the short term, but it will also force enterprises to produce with lower inputs and improve production efficiency. In the long term, such transformation and innovation may eliminate inefficient enterprises and bring technological breakthroughs and competitive advantages in the market, and those enterprises that survive the ban will not only be able to reduce their reliance on resources, but also be able to provide products and services of higher quality, thus competing in the increasingly competitive market, thereby standing out in an increasingly competitive market, and research shows that technological innovations decrease emissions substantially in the long term [54]. Accordingly, the following hypotheses are formulated.

H2 the ban on “foreign waste” would increase GTFP in manufacturing through innovation incentives

### 3.3 Resource allocation effects

As a kind of environmental regulation, the ban on “foreign waste” can also play a significant resource allocation effect in the process of implementation. First of all, the rising cost of raw materials will prompt enterprises to improve the allocation and use of resources to maintain their production efficiency, and the implementation of the ban will also eliminate the inefficient allocation of resources, forcing them to withdraw from the market, thus fostering manufacturing development. Secondly, most of the imported solid wastes are from complex sources with high pollution content, and often produce a large amount of pollution in the process of recycling and treatment, while the implementation of the ban reduces the use of solid wastes from unknown sources and increases the recycling of domestic solid wastes, which directly leads to the reduction of pollution in the production of manufacturing enterprises, thus improving the GTFP and promoting the greening of manufacturing. Finally, the ban on “foreign waste” prompts manufacturing enterprises to reconfigure their production factors and change their product mix by reducing the production of products that depend on imported solid waste, thereby reducing production pollution. Accordingly, the following assumptions are made.

H3 the ban on “foreign waste” would increase GTFP in manufacturing by improving resource allocation

In sum, although the ban on “foreign waste” may have put short-term cost pressure on the manufacturing sector, it has also created opportunities for firms to improve their technology, resource allocation, market positioning, and long-term competitiveness, so this paper will test the above hypotheses while assessing the combined effects of the policy.

## 4 Data and methodology

### 4.1 Sample selection and data sources

Select 30 provincial manufacturing panel data from China from 2010 to 2020 as sample. The data required for calculating GTFP of the manufacturing industry come from China Industrial Statistics Annual, the China Environmental Statistics Annual and the provincial statistical annual reports, the data on treatment intensity (treat) come from the statistics of the General Administration of Customs and the statistical database of the National Research Network, the data related to control variables come from China Statistical almanac, China Science and Technology Statistics almanac, The Price Statistics almanac of China, China Industrial Statistics Annual, National Bureau of Statistics and provincial statistical annual reports.

### 4.2 Description of variables

#### 4.2.1 Explained variables

GTFP of the manufacturing industry is the explanatory variable, referring to authoritative studies [55,56], we calculate it by applying the EBM-GML index method through MATLAB software. SBM belongs to the non-radial model, which can't deal with the radial problem, while the EBM model, as a mixed-distance model containing both radial and non-radial possibilities, can overcome the above shortcomings, so the efficiency value calculated by using the EBM model is used to represent GTFP. The ML index doesn't have cyclicity and comparability. Therefore, the GML index constructed based on the common frontier surface is used, and then it is decomposed into technical progress (TC) and efficiency improvement (EC), and the results are analysed further in depth through its change trend.

#### 4.2.2 Core explanatory variables

The policy shock is the core explanatory variable, represented by the cross-multiplier of the treatment intensity and the time dummy variable, where the treatment intensity is constructed by multiplying 10,000 and taking the logarithmic construct of the share of the value volume of solid waste in raw materials and intermediate products imported by the industry, generating the time dummy variable, which is taken to be 1 if the year is greater than 2017, and 0 otherwise.

#### 4.2.3 Control variables

Checking the literature related to environmental regulation and GTFP, to ensure the accuracy of the results, five indicators, namely, gross regional product, foreign direct investment, energy price index, industrial agglomeration level, and environmental regulation index, were selected as control variables. Among them, GDP and FDI use CPI to deflate with 2010 as the base period; in addition, in order to make the effect of the ban have a certain degree of differentiation from other environmental policies, the strength that other environmental rules have is also added for control, which is reflected in the proportion of the investment in the completion of the industrial pollution control to the industrial value-added, to exclude the interference of other environmental regulation type of policies to a certain extent.

#### 4.2.4 Mechanism variables

Based on the above theoretical analysis, the ban on “foreign waste” will work on GTFP through cost effect, innovation incentive effect, and resource allocation effect at the same time. The cost effect of the ban is mainly through the cost of raw materials, and data on the proportion of solid waste in raw materials of manufacturing enterprises are not available, so the general industrial solid waste utilization rate is chosen as an indicator to measure this initially.

Regarding the incentive effect of innovation, the existing literature mostly measures it from the output side through the number of patents of enterprises, but this approach is not suitable for this study: on the one hand, the ban was fully implemented only in 2018, which is a relatively short time dimension, and it is difficult for patents to change significantly as the results of innovation in a short period; on the other hand, the complexity of the manufacturing industry production, technology and other characteristics of the output of patents takes a longer period. Therefore, this paper starts from the input and output sides at the same time, based on the availability of data, reference to related research, and select the internal expenditure of R&D funds of industrial enterprises as the measurement index on the input side [57,58]. On the output side, we choose the amount of patent applications for inventions by industrial enterprises above a certain size to measure the substantive innovation of enterprises [59]. In addition, considering that some new products do not apply for patents, this paper also selects the output value of new product sales as a measurement index, so as to make a more comprehensive evaluation of the technological innovation efficiency of industries in each province from the aspects of competitiveness and economic effect of industrial enterprises [60].

The firm-level resource allocation effects of the ban are mainly reflected in the short term through the optimization of firms' production lines, for which data are difficult to obtain. Therefore, this paper analyses the firm-level resource allocation effects of the ban at the industry level through the relevant decomposition term of GTFP, EC (Efficiency Improvement), which serves as a decomposition term that separates the technological progress element of the GTFP. Some studies have also separated EC to measure efficiency improvement separately [61]. EC as a decomposition term can separate the technological progress element of green TFP, and some studies have also separated EC from efficiency improvement to measure it separately. Table 1 contains the variables details.

**Table 1:** Definition of variables.

property	name	meaning	calculation method
input	L	labor input	Average number of workers in the manufacturing sector (10,000 RMB)
	K	capital input	Capital stock of manufacturing companies exceeding the specified size, based on the perpetual inventory method and the depreciation rate of 9.6 %.
	E	energy input	Industrial end-use energy consumption (tonnes of standard coal)
output	EP	expected outputs	Manufacturing output deflated to a 2010 base period using the industrial producer price index (100 million RMB)
	UEP	unexpected outputs	Industrial emissions = industrial sulphur dioxide emissions + industrial nitrogen outputs oxide emissions + industrial solid particulate emissions (tonnes)
			Industrial wastewater = total
			industrial COD discharge COD + total industrial ammonia nitrogen discharge (tonnes)
explained variables	gtfp	GTFP	Efficiency values calculated by the EBM method
Core explanatory variable	DID	policy shock = treat × post	The cross-multiplier of treat and post, treat = ln (10000∗value of solid waste imports/value of industrial raw material imports in 2017),post takes 1 for year>2017, otherwise 0
Mechanism variables	was_u	Industrial solid waste utilization rate	Comprehensive use of general industrial solid waste/General industrial solid waste generation (tonnes)
	ln*rd*	Internal expenditure on R&D	Logarithmic treatment of internal (10,000 RMB) expenditures on R&D
	ipat	Amount of patent applications	Amount of patent applications by industrial companies above specific level (10,000)
	npro	value of new products	Revenue from sales of new products by industrial enterprises above designated size, deflated using the industrial producer ex-factory price index with 2010 as the base period (100 million RMB)
control variables	ln*gdp*	GDP	Logarithmic treatment of GDP (100 million RMB)
	ln*fdi*	foreign direct investment	Logarithmic treatment of FDI (100 million RMB)
	ener*_*p	The index of energy price	The index of fuel and power price
	enter*_*clus	level of industrial clustering	Regional entropy method: (urban manufacturing workers in each industrial region in the year/total urban agglomeration workers in the region in the year)/(urban manufacturing workers in the country in the year/total urban workers in the country in the year)
	en*_*c	The index of environmental regulation	Environmental Completed investment in industrial pollution control/industrial value added

### 4.3 Calculation of GTFP in manufacturing industry

The EBM model combined with the GML index was used to measure GTFP in manufacturing and further decomposed into technical progress (TC) and efficiency improvement (EC), the reasons why we chose this calculation method have been explained in the variable description section. The specific model is as follows:

$$\gamma =\min\frac{\theta -\varepsilon_{x}\sum_{i=1}^{m}\frac{\omega_{i}^{-}s_{i}^{-}}{x_{ik}}}{\varphi +\varepsilon_{y}\sum_{r=1}^{l}\frac{\omega_{r}^{+}s_{r}^{+}}{y_{rk}}+\varepsilon_{z}\sum_{t=1}^{p}\frac{\omega_{t}^{-}s_{t}^{-}}{z_{tk}}}$$

(1) $$s.t.\left\{ \begin{array}{c} X\delta +s_{i}^{-}=\theta x_{k},i=1,2,\cdots ,m\\ Y\delta -s_{r}^{+}=\varphi y_{k},r=1,2,\cdots ,l\\ Z\delta +s_{t}^{+}=\varphi z_{k},t=1,2,\cdots ,p\\ \delta >0,s_{i}^{-},s_{r}^{+},s_{t}^{-}\geq 0 \end{array}\right.$$

In equation (1), X, Y, and Z denote m inputs, l expected outputs and p unexpected outputs in the manufacturing production process, respectively; k is the number of decision units; γ (0 ≤γ≤1) is the optimal efficiency value; $\omega_{i}^{-}，\omega_{r}^{+}\;and\;\omega_{t}^{-}$ denote the weights of the i-th input, the r-th output, and the t-th unexpected outputs, $s_{i}^{-}，s_{r}^{+}\;and\;s_{t}^{-}$ denote the relaxation variables for the i-th input, the r-th output, and the t-th unexpected outputs, and ε(0≤ε≤1) is the significant parameter that combine radial efficiency value θ and the nonradial relaxation variables.

Construct the GML index and decompose it as follows:

(2) $${GML}^{t,t+1}\left(x^{t},y^{t},z^{t},x^{t+1},y^{t+1},z^{t+1}\right)=\frac{E^{g}\left(x^{t+1},y^{t+1},z^{t+1}\right)}{E^{g}\left(x^{t},y^{t},z^{t}\right)}=\frac{E^{t+1}\left(x^{t+1},y^{t+1},z^{t+1}\right)}{E^{t}\left(x^{t},y^{t},z^{t}\right)}\times \left(\frac{E^{g}\left(x^{t+1},y^{t+1},z^{t+1}\right)}{E^{t+1}\left(x^{t+1},y^{t+1},z^{t+1}\right)}∙\frac{E^{t}\left(x^{t},y^{t},z^{t}\right)}{E^{g}\left(x^{t},y^{t},z^{t}\right)}\right)=EC\times TC$$

E in equation (2) denotes the efficiency value for each period calculated by the EBM model, and EC and TC are the technical efficiency change and technical change from period t to t+1, respectively.

### 4.4 Econometric model

Since the foreign waste ban is a national policy, implemented nationwide, the traditional Difference-in-Differences (DID) model with clear experimental and control groups cannot be used, however, the intensity of policy shocks varies across regions. Therefore, we conducted this study using a continuous Difference-in-Differences model constructed with continuous TREAT variable, and the method of constructing continuous TREAT variable is shown in the variable description section above.

To test the policy effect, this paper constructs the following econometric model:

(3) $${GTFP}_{it}=\alpha +\beta {DID}_{it}+\phi X_{it}+\eta_{i}+\lambda_{t}+\varepsilon_{it}$$

In equation (3), i represents province, t represents year, GTFP is the GTFP of province i in year t, DID is the cross-multiplier of the treatment intensity and policy shock time dummy variables, and X is the control variables, the η, and λ denote individual and time fixed effects, respectively. Based on the exclusion of interference from environmental regulatory policies that exert similar effects through the above environmental regulatory indices, co-temporal factors such as other policy shocks that occur at the national level are further controlled for through the control of time fixed effects [62]， ε is the random error term, α is the intercept term, β is the coefficient of the policy effect, and ϕ is the coefficients of the control variables

## 5 Empirical results and analysis

### 5.1 Descriptive statistics

The description of data is shown in Table 2. The average level of GTFP in 30 provinces from 2010 to 2020 is about 0.47, distributed in 0.18–1, with overall fluctuations, and is mainly characterized by greater fluctuations in EC. The average level of cross-multiplier term DID, which is policy shocks suffered by the regions, is about 1, distributed in 0–7.6525, and with similarly high fluctuations. The values of the remaining variables are also consistent with their economic meanings.

**Table 2:** Descriptive statistics of variables.

Variables	N	Mean	Std. Dev	Min	Max
gtfp	330	0.4741	0.1515	0.1848	1
ec	330	1.0073	0.1126	0.4423	1.7359
tc	330	1.0449	0.0915	0.8503	1.6354
DID	330	0.9903	2.1619	0	7.6525
was_u	330	65.3550	19.2260	5.6251	99.8288
ln*rd*	330	4.8927	1.3627	0.6062	7.5640
ipat	330	0.8907	1.5867	0.0037	12.7497
npro	330	0.5170	0.7466	0.0007	4.3789
ln*gdp*	330	9.5951	0.8824	7.0424	11.3586
ln*fdi*	330	3.3572	1.7834	0	5.9014
ener*_*p	330	1.0391	0.1109	0.6172	1.4435
enter*_*clus	330	0.8319	0.3465	0.2921	1.8244
en*_*c	330	0.0029	0.0028	0.0000	0.0245

**Fig. 3:**
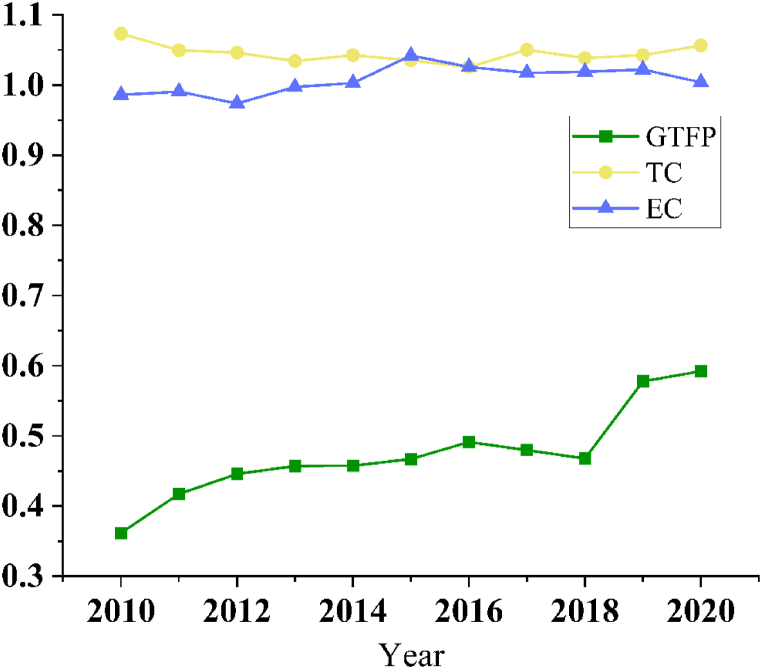
Graph of efficiency trends.

Fig. 3 shows the trends of GTFP, technological progress, and efficiency improvement in 30 provinces. From the figure, it can be seen that from 2010 to 2020, the GTFP of the 30 provinces generally shows a fluctuating upward trend, in which Beijing and the coastal areas such as Shanghai and Tianjin have increased more, indicating that they have achieved relatively good results in the greening of their manufacturing industries in recent years.

**Fig. 4:**
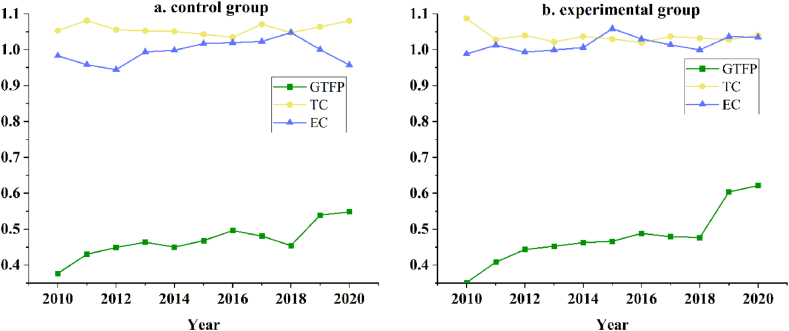
Plot of efficiency change after grouping.

Fig. 4 divides the 30 provinces into 2 groups based on the median sample size of treatment intensity, showing the trends of their GTFP, technological progress, and efficiency improvement, respectively.

From the above trend graph, it can be seen that before and after the release of the ban on “foreign waste”, although the GTFP of both groups increased, the experimental group's increase was greater, in which the EC of the experimental group had an upward trend, while the control group had the opposite; the TC of both groups had an upward trend, but in the year 2018, the TC of the control group grow slower, so the ban may promotes technological progress, which will be further tested in subsequent research; however, due to the above data availability reasons, it is difficult to test H3 in depth, and therefore can only be preliminarily speculated that the existence of the resource allocation effect of the ban through the different trends in EC here, to be improved in the subsequent research to be further Exploration.

### 5.2 Parallel trend test

The confidence interval method was used to test for parallel trends using 2016 as the base year.

**Fig. 5:**
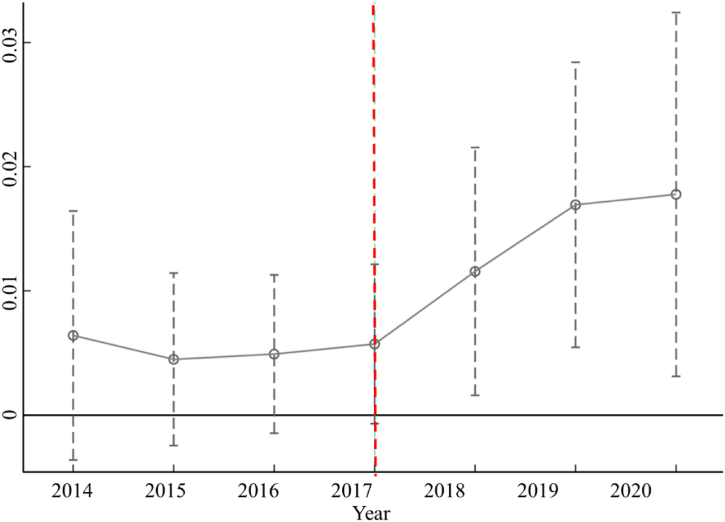
Parallel trend test.

From Fig. 5 we can see that the coefficient before the implementation of the policy is maintained near 0, indicating that the trends of both groups are basically the same, and the parallel trend test is passed. After 2018, the coefficient has an upward trend, indicating that the ban on “foreign waste” is likely to promotes GTFP of the manufacture industry.

### 5.3 Benchmark regression

Table 3 shows the results of the benchmark regression of GTFP of the manufacturing industry in 30 provinces for the ban on “foreign waste”. Model (4) is the result of adding control variables, time fixed effects and individual fixed effects at the same time, model (3) removes the time fixed effects on this basis, model (2) removes the control variables based on model (4), and model (1) removes the time fixed effects and control variables at the same time.

**Table 3:** Results of benchmark regression.

Variables	(1)	(2)	(3)	(4)
DID	0.0115∗∗∗ (0.0041)	0.0114∗∗∗ (0.0036)	0.0125∗∗∗ (0.0039)	0.0126∗∗∗ (0.0036)
ln*fdi*			0.0237∗∗∗	0.0265∗∗∗
			(0.0060)	(0.0069)
ln*gdp*			0.209∗∗∗	0.142∗
			(0.0250)	(0.0742)
ener_p			−0.0036	−0.0022
			(0.0023)	(0.0021)
enter_clus			−0.0726	−0.0745
			(0.0546)	(0.0524)
en_c			1.057	1.253
			(1.648)	(1.592)
_cons			−1.557∗∗∗	−0.926
			(0.259)	(0.713)
Individual fixed effect	YES	YES	YES	YES
Time fixed effect	NO	YES	NO	YES
N	330	330	330	330
Adjusted-R^2^	0.6750	0.7601	0.7398	0.7757
F	28.76	10.24	29.89	8.63

Note: ∗∗∗, ∗∗, and∗ denote significance levels of 1 %, 5 %, and 10 %, and robust standard errors are shown in parentheses, same below.

From the above results, it can be seen that the ban on “foreign waste” has promoted the GTFP of the manufacturing industry, in model 4 we can see that for every 1 increase in policy impact strength (DID), GTFP increases by an average of 0.0126, holding all other conditions constant. This is consistent with the results obtained in the findings of current researches that focus on the relationship between the environmental legislation and the GTFP of manufacturing industry [63,64], which shows that the positive effect of its innovation effect and resource allocation effect is higher than the negative effect of the cost effect. There may be two main reasons for this result: firstly, due to the improvement of China's independent research and development capacity in recent years, enterprises have been able to carry out innovative activities in a more convenient way. Secondly, because of the increasing dynamism of the market, the ability of resource reallocation has been greatly improved. Based on this result, China should further implement the foreign waste ban in the future, in order to continue to leverage the positive impact of this policy on the green development of the manufacture sector, and to contribute to the green growth. But due to the short year of implementation of the policy, it is difficult to observe its long-term effect, which needs to be further analysed in the future.

### 5.4 Heterogeneity analysis

The heterogeneity test is conducted from three aspects: region, industrial structure, and the development level of the manufacturing sector. Restricted by resource endowment, economic foundation and other reasons, China's regions in the economic and social and other aspects of the phenomenon of unbalanced and insufficient development, inland areas in the industrial structure, green infrastructure, etc. lag behind the coastal areas, so the role of the policy there is a potential heterogeneity, in order to test the existence of such a heterogeneity, with reference to the study of Zhang [65] In order to test whether such heterogeneity exists. In Table 4, the sample is divided into 2 groups: coastal and inland, where (1) is the coastal region and (2) is the inland region; in addition, the heterogeneity test is also conducted to further analyse the differences in economy structure and the manufacturing sector's level of greening: the industrial structure and the level of development are divided into two groups based on the median, where (3) is the region where the manufacturing industry accounts for the higher percentage of GDP, and (5) is the region where the level of GTFP is the higher.

**Table 4:** Heterogeneity test regression results.

Variables	Regional		Structure		Developmental	
	(1)	(2)	(3)	(4)	(5)	(6)
DID	0.0206∗∗ (0.0099)	0.0110∗∗ (0.0051)	0.0060 (0.0079)	0.0099∗∗ (0.0045)	0.0189∗∗∗ (0.0049)	−0.0026 (0.0043)
_cons	−1.1898 (1.2519)	−0.6434 (0.8803)	−4.8752∗∗∗ (0.7968)	1.8198 (1.2098)	2.6393 (1.7692)	−1.3292∗∗∗ (0.5040)
Control variables	YES	YES	YES	YES	YES	YES
Individual fixed effect	YES	YES	YES	YES	YES	YES
Time fixed effect	YES	YES	YES	YES	YES	YES
N	121	209	165	165	165	165
Adjusted-R^2^	0.7713	0.7546	0.8301	0.8544	0.7621	0.6505
F	4.30	6.10	16.76	6.95	5.37	4.54

From the above results, it can be seen that the ban has a more significant positive impact on coastal areas, areas with a lower share of manufacturing industries, and areas with a higher level of development. This may be because coastal areas tend to be more technology-intensive, with a higher level of green development and more efficient use of resources, and the recycling industry in coastal areas is already relatively developed, with a high utilization rate of local solid waste recycling, and the cheap raw material sources of manufacturing enterprises do not have a high degree of reliance on imported solid wastes, so the ban brings about a smaller impact on the cost effect, and a more open and active market environment makes the incentive effect and the resource allocation effect of the ban better exerted; while for the lower proportion of manufacturing industries, the ban has a more significant positive impact on. The more open and active market environment makes innovation incentives and resource allocation effects better utilized; for regions with a lower proportion of manufacturing and a higher level of green development, their industrial structure is more diversified, and the development of the recycling industry and other service industries is more favourable, so the cost impact of the ban is smaller, and due to the lower reliance on the manufacturing industry, the level of pollution in these regions is usually lower, and the local community pays more attention to green development and the protection of the ecological environment. In addition, due to their lower dependence on manufacturing, these regions are usually less polluted and will pay more attention to green development and ecological protection, so the original level of green development will be higher, and the corresponding policies and systems in terms of infrastructure and green recycling economy will be more perfect, so the innovation incentives and resource allocation effects generated by the ban can be better utilized. From another dimension, the coastal area is often the area with lower manufacturing weight and higher level of green development, and the three are unified, which to a certain extent ensures the credibility of the heterogeneity test.

### 5.5 Mechanism test

#### 5.5.1 Cost effects

From the above analysis, the recycling industry largely affects the effect of the policy. For regions with a lagging recycling industry, the ban on “foreign waste” has caused a large number of low-end recycling enterprises to be impacted and face the risk of closure, thus further reducing the source of cheap materials and magnifying the negative impact of the ban; however, for regions with a well-developed recycling industry, the local waste classification work is more perfect, so the recycling enterprises can make more use of local solid waste and the raw materials for processing are somewhat guaranteed. However, for regions with a higher level of development of the recycling industry, the local waste classification and recycling work is more perfect, so renewable resources processing and utilization enterprises can make more use of local solid wastes, and the raw materials for processing are guaranteed to a certain extent, so they are subjected to a smaller negative impact.

To verify the above mechanism, a regression on the utilization rate of general industrial solid waste generation in 30 provinces by the ban on “foreign waste” was conducted according to the regional grouping of the heterogeneity test to conduct a preliminary test. In Table 5, Table 1 is the coastal area and (2) is the inland area. From the results in the table, the ban significantly reduced the utilization rate of industrial solid waste in non-coastal areas, while the utilization rate of industrial solid waste will be positively correlated with the GTFP from the aspects of input costs and non-expected outputs, which is also in line with the results of the heterogeneity test in which the ban has a more significant positive impact on the coastal areas, which verifies the mechanism mentioned above to a certain extent, and also The cost effect of the ban is verified, and hypothesis 1 is valid. In response to this situation, we suggest that the Chinese Government should pay more attention to the development of the recycling industry, vigorously develop the circular economy, and replace imported solid wastes with domestic solid wastes that have been better recycled and treated, so as to mitigate the negative impact of this cost effect.

**Table 5:** Results of the mechanism test.

Variables	Cost effects		Innovation incentive effects			Resource allocation effects
	(1)	(2)	(3)	(4)	(5)	(6)
DID	−0.0237 (0.7679)	−0.8553∗∗ (0.3618)	0.0272∗∗∗ (0.0073)	0.1119∗∗∗ (0.0245)	0.0452∗∗∗ (0.0107)	−0.0013 (0.0048)
_cons	407.4207∗∗∗ (111.9471)	−304.7108∗∗∗ (55.6158)	−14.2673∗∗∗ (1.5452)	−16.3757∗∗∗ (4.4608)	−5.7147∗∗∗ (1.6829)	0.2041 (0.7562)
Control variables	YES	YES	YES	YES	YES	YES
Individual fixed effect	YES	YES	YES	YES	YES	YES
Time fixed effect	YES	YES	YES	YES	YES	YES
N	121	209	330	330	330	330
Adjusted-R^2^	0.9312	0.7922	0.9905	0.8916	0.9001	0.2034
F	3.07	12.70	60.20	8.54	8.85	1.11

#### 5.5.2 Innovation effects

Models (3), (4), and (5) in Table 5 show the impact of the ban on industrial enterprises' R&D investment, the number of invention patent applications, and the output value of new product sales, respectively. As can be seen from the table, the ban significantly increases innovation of industrial enterprises measured in three ways, indicating that the policy creates innovation incentives for enterprises, although the rise in the cost of enterprise innovation investment may squeeze its production and operation funds, but at the same time this will bring new technologies and new products, improve efficiency and competitiveness, and promote the optimization of the entire industry, in addition, the increase in the cost of R&D also forces the enterprises to optimize their production lines, improve the efficiency of resource utilization, and strengthen the resource allocation effect, thus having a positive effect on manufacturing GTFP. The above mechanism verifies the innovation incentive effect of the ban, and hypothesis 2 is established. Therefore, in order to better utilise this innovation effect, the Chinese government should further improve the raw material market to help enterprises find suitable alternative materials faster, and at the same time give support to enterprises with outstanding innovation to further stimulate the innovation activities of manufacturing firms.

#### 5.5.3 Resource allocation effects

Model (6) in Table 5 shows the effect of the ban on manufacturing EC in each region. As can be seen from the table, the ban did not affect efficiency improvements in the manufacturing sector, and it is surmised that the positive impact of the ban was mainly through the innovation incentive effect. This result may be due to the fact that, on the one hand, the barriers to entry and exit in the manufacturing sector are relatively high, making it difficult in the short term to improve the efficiency of resource allocation by eliminating backward firms and bringing in highly efficient firms, besides, China's market system, although it has been making progress, is not perfect, so it is difficult to significantly affect the resource allocation. Therefore, to address this situation, we suggest that the Chinese government should provide more policy or financial support to enterprises with high technology level to help them enter the market and compete effectively, and at the same time, further improve the financial market to better promote the circulation of funds and resources, so as to better utilise the resource allocation effect of the ban on foreign waste.

### 5.6 Robustness tests

#### 5.6.1 Variable substitution

In many imported solid wastes, the proportion of waste paper is the highest, data show that in 2015 the proportion of waste paper in the national imported waste was as high as 62.1 %, and according to the previous analysis, China's paper industry is particularly dependent on the import of waste paper, and many current studies on the effect of the ban on “foreign waste” are paper industry as an example [41,50,66,67]. It can be seen that the paper industry is an important representative of the manufacturing industry affected by the ban. Therefore, this paper to 30 provinces in 2016, paper and paper products industry on the overall industrial sales value of the manufacturing industry as a replacement variable, indicating the size of different regions by the degree of the role of the policy, the results are shown in Table 6, still shows a significant positive effect, to a certain extent, to verify the robustness of the results.

**Table 6:** Robustness test results.

variables	Substitution of variable	Reduced study period	shrinkage test	
			1 % level	5 % level
DID	0.0274∗∗∗ (0.0094)	0.0112∗∗∗ (0.0041)	0.0126∗∗∗ (0.0035)	0.0129∗∗∗ (0.0036)
_cons	−0.6853 (0.7136)	−1.4202∗∗ (0.6723)	−0.9264 (0.7132)	−0.9239 (0.7131)
Control variables	YES	YES	YES	YES
Individual fixed effect	YES	YES	YES	YES
Time fixed effect	YES	YES	YES	YES
N	330	330	330	330
Adjusted-R^2^	0.7704	0.8069	0.7757	0.7760
F	6.13	6.00	8.63	8.67

#### 5.6.2 Reduced sample period

The new crown epidemic in 2020 had an immeasurable impact on all economic and social sectors in China, so to exclude the possible impact of the epidemic on the results; this paper removes the data for 2020 and adjusts the sample period to 2010–2019 to reassess the effect of the ban. The results, as shown in Table 6, show a significant positive effect of the policy and the results remain robust.

#### 5.6.3 Extreme value processing

In order to avoid the possible bias of the results from extreme values, the core explanatory variables were subjected to two-way tailoring at the 1 % and 5 % levels, respectively. As can be seen from Table 6, the results remain significant after two different levels of shrinkage treatment, which ensures the robustness of the results to a certain extent.

#### 5.6.4 Placebo testing

**Fig. 6:**
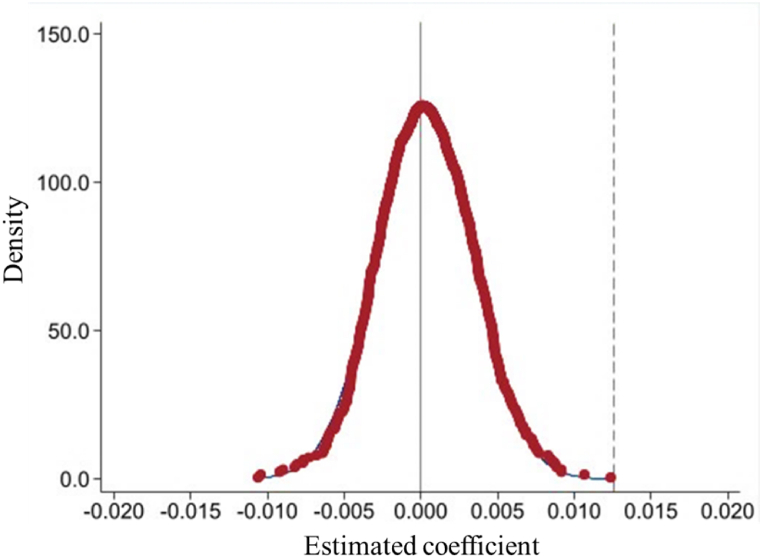
Plot of placebo test regression coefficients.

The interaction term is randomly selected 1000 times to be brought into the benchmark regression model (4), i.e., the placebo test is conducted by randomly constructing the experimental group. As can be seen in Fig. 6, the regression coefficients after random sampling are centrally distributed around 0, while the estimated coefficients of the baseline regression turn out to deviate significantly from the distribution of the test, suggesting that the effects of other policy and random factors can be ruled out, and that the placebo test passes, suggesting that the results are robust to some degree.

## 6 Conclusions and policy implications

In order to mitigate the negative impacts of growth and thus achieve high-quality economic development, China introduced the ban on “foreign waste” in 2017. In order to explore the actual effect of the ban and assess the policy effect, this paper use the Continuous DID model, takes the 30 provincial manufacturing panel data from China from 2010 to 2020 as sample, and makes a comprehensive assessment of the policy effects of China's ban on “foreign waste” on the manufacturing industry through GTFP based on the DID method, we get following conclusions: Firstly, the ban on “foreign waste” introduced in 2017 contributes to promoting GTFP overall of the manufacturer's sector. Secondly, the ban has both positive and negative implications for Chinese manufacturing industry due to the innovation incentive effect and the cost effect, while the resource allocation effect has not been well utilized. Thirdly, the effect of the ban is somewhat heterogeneous, with a more significant positive impact on coastal areas, areas with a lower share of manufacturing, and areas with a higher level of green development.

### 6.1 Policy recommendations

Firstly, raise residents' awareness of waste recycling, promote the recycling of self-produced waste in the manufacturing sector through agglomeration development, and mitigate the cost effect of the ban through the improvement of domestic solid waste resources. The government should increase publicity efforts and take appropriate incentives to encourage residents to carry out the categorisation of waste, and at the same time, establish manufacturing industry parks to gather solid waste produced by enterprises, promote its recycling and ultimately build a sustainable supply chain, and the restructuring of the industry can be achieved accompanied by reduction of the negative impacts brought by the ban.

Secondly, through the relevant policies to stimulate innovation, guide the flow of factors, strengthen the incentive effect of innovation while playing out the policy should be the effect of resource allocation. The government should further strengthen the positive effect through tax incentives and other sustained and stable measures, while guide the resources to flow to more efficient companies through the improvement of the financial market, raw material market, etc., and give financial support to more efficient and innovative enterprises through the establishment of a development fund and other measures to promote the improvement of the Manufacturing structure.

Thirdly, according to regional heterogeneity, targeted measures should be taken. Strengthen international cooperation and exchanges for coastal regions to further improve environmental protection and promote their governance experience and related technologies; for inland regions, focus on renewable resources, and provide greater possibilities for the development of recycling economy, and technological research and development through a diversified industry system to promote the environmentally friendly development of the manufacturing sector; in addition to further opening up the inter-regional raw materials market and promoting resource allocation, and optimizing the manufacturing structure. In addition, it can further open up the inter-regional raw material market and promote the cross-regional circulation of resources.

## Funding

This research is funded by Lanzhou University Key Project of Educational and Teaching Reform Research (202202) ：Innovation and Practice in the Training Model for Top-Notch Interdisciplinary Economics Talents under the Background of the New Liberal Arts.

## Data availability statement

The data will be available on request from corresponding author.

## CRediT authorship contribution statement

**Yanting Li:** Writing – original draft, Validation, Funding acquisition, Formal analysis, Data curation. **Xiaozhe Li:** Writing – review & editing, Writing – original draft, Validation, Methodology, Formal analysis. **Fayyaz Ahmad:** Writing – review & editing, Writing – original draft, Conceptualization.

## Declaration of competing interest

The authors declare that they have no known competing financial interests or personal relationships that could have appeared to influence the work reported in this paper.

## References

[1] Kirikkaleli D. (2020). New insights into an old issue: exploring the nexus between economic growth and CO2 emissions in China. Environ. Sci. Pollut. Control Ser..

[2] Wu G., You D. (2019). The influence mechanism of environmental regulation on technology innovation and green total factor productivity : based on the moderating effect of fiscal decentralization. Journal of Industrial Engineering and Engineering Management.

[3] Ahmed Z., Asghar M.M., Malik M.N., Nawaz K. (2020). Moving towards a sustainable environment: the dynamic linkage between natural resources, human capital, urbanization, economic growth, and ecological footprint in China. Resour. Pol..

[4] Xue W., Liu J. (2010). Environmental regulation and its evaluation in China. China Pop. Resour. Environ.

[5] Yu Y., Yin L. (2022). The evolution of Chinese environmental regulation policy and its economic effects: a summary and prospect. Reform.

[6] Geng Y., Sarkis J., Ulgiati S., Zhang P. (2013). Measuring China's circular economy. Science.

[7] Stahel W.R. (2016). The circular economy. Nature.

[8] Qu S., Guo Y., Ma Z., Chen W.-Q., Liu J., Liu G., Xu .M. (2019). Implications of China's foreign waste ban on the global circular economy. Resour. Conserv. Recycl..

[9] Haoyu X., Yang W., Junshi W., Ruyue Z. (2022). Research on impact of China's ban on the import of foreign garbage on global solid waste treatment. Industrial Safety and Environmental Protection.

[10] Yamashita K., Yamamoto N., Mizukoshi A., Noguchi M., Ni Y., Yanagisawa Y. (2009). Compositions of volatile organic compounds emitted from melted virgin and waste plastic pellets. J. Air Waste Manag. Assoc..

[11] Tsai C.-J., Chen M.-L., Chang K.-F., Chang F.-K., Mao I.-F. (2009). The pollution characteristics of odor, volatile organochlorinated compounds and polycyclic aromatic hydrocarbons emitted from plastic waste recycling plants. Chemosphere.

[12] Atkinson R. (2000). Atmospheric chemistry of VOCs and NOx. Atmos. Environ..

[13] Li J., Takeuchi K. (2023). Import ban and clean air: estimating the effect of China's waste import ban on ozone pollution. Environ. Econ. Pol. Stud..

[14] Zhang X., Chen X., Zhang X. (2018). The impact of exposure to air pollution on cognitive performance. Proc. Natl. Acad. Sci. USA.

[15] He J., Liu H., Salvo A. (2019). Severe air pollution and labor productivity: evidence from industrial towns in China. Am. Econ. J. Appl. Econ..

[16] Heyes A., Zhu M. (2019). Air pollution as a cause of sleeplessness: social media evidence from a panel of Chinese cities. J. Environ. Econ. Manag..

[17] Huang Q., Chen G., Wang Y., Chen S., Xu L., Wang R. (2020). Modelling the global impact of China's ban on plastic waste imports. Resour. Conserv. Recycl..

[18] Brooks A.L., Wang S., Jambeck J.R. (2018). The Chinese import ban and its impact on global plastic waste trade. Sci. Adv..

[19] Yoshida A. (2022). China's ban of imported recyclable waste and its impact on the waste plastic recycling industry in China and Taiwan. J. Mater. Cycles Waste Manag..

[20] Shen H., Zhou Y. (2017). Supervision of environmental policy enforcement and firm environmental performance: evidence from a quasi-natural experiment. Nankai Business Review.

[21] Ming L., Xiaowen Y. (2013). Local fiscal expenditure, environmental regulation and low-carbon economic transformation in China. Economic Science.

[22] Shen N., Liu F. (2012). Can intensive environmental regulation promote technological innovation? Porter hypothesis reexamined. China Soft Science.

[23] Chen S. (2010). Energy-save and emission-abate activity with its impact on industrial win-win development in China: 2009–2049. Econ. Res. J..

[24] Du L., Zhao Y., Tao K., Lin W. (2019). Compound effects of environmental regulation and governance transformation in enhancing green competitiveness. Econ. Res. J..

[25] Porter M.E., Linde C.v. d. (1995). Toward a new conception of the environment-competitiveness relationship. J. Econ. Perspect..

[26] Xiaodong L., Zhen L., Panpan Z. (2022). Environmental Regulation,Media attention and green total factor productivity:an analysis based on provincial panel data. Ecol. Econ..

[27] Yi L., Xu Y., Maoxing H. (2020). Environmental regulation and green total factor productivity——analysis of the mediating effect based on different technological progress path. Contemporary Economic Management.

[28] Shen C., Zheng J. (2021). Environmentanterprl Ｒegulations，Firm's dynamics and total factor productivity in manufacturing: an empirical analysis using industrial Eises'Pollution emissions data. Nanjing Journal of Social Sciences(03).

[29] Ramzan M., Hossain M.R., Abbasi K.R., Adebayo T.S., Alvarado R. (2024). Unveiling time-varying asymmetries in the stock market returns through energy prices, green innovation, and market risk factors: wavelet-based evidence from China. Econ. Change Restruct..

[30] Abbasi K.R., Zhang Q., Ozturk I., Alvarado R., Musa M. (2024). Energy transition, fossil fuels, and green innovations: paving the way to achieving sustainable development goals in the United States. Gondwana Res..

[31] Zheng L., Abbasi K.R., Salem S., Irfan M., Alvarado R., Lv K. (2022). How technological innovation and institutional quality affect sectoral energy consumption in Pakistan? Fresh policy insights from novel econometric approach. Technological Forecasting and Social Change.

[32] Abbasi K.R., Zhang Q., Alotaibi B.S., Abuhussain M.A., Alvarado R. (2024). Toward sustainable development goals 7 and 13: a comprehensive policy framework to combat climate change. Environ. Impact Assess. Rev..

[33] Iqbal N., Abbasi K.R., Shinwari R., Guangcai W., Ahmad M., Tang K. (2021). Does exports diversification and environmental innovation achieve carbon neutrality target of OECD economies?. J. Environ. Manag..

[34] Wang C., Abbasi K.R., Irfan M., Ben-Salha O., Bandyopadhyay A. (2024). Navigating sustainability in the US: a comprehensive analysis of green energy, eco-innovation, and economic policy uncertainty on sectoral CO2 emissions. Energy Rep..

[35] Albaker A., Abbasi K.R., Haddad A.M., Radulescu M., Manescu C., Bondac G.T. (2023). Analyzing the impact of renewable energy and green innovation on carbon emissions in the MENA region. Energies.

[36] Zhang T. (2014). Research on green innovation incentive of environmental regulation.

[37] Wenwen J., Desheng L. (2018). Human capital allocation and China 's innovation performance. Econ. Perspect..

[38] Hsieh C.-T., Klenow P.J. (2009). Misallocation and manufacturing TFP in China and India. Q. J. Econ..

[39] Hanyue X., Hui S., Hui W., Long x. (2022). Innovation or resource reallocation : how does environmental regulation affect total productivity growth. Ecol. Econ..

[40] Zhou Y., Guo Q., Zou D. (2022). Environmental regulation and product mix of firm—evidence from multi-product exporters in China. China. Ind. Econ.

[41] Bo L. (2020). Study on the environmental and economic impact of the waste imported ban. https://d.wanfangdata.com.cn/thesis/ChJUaGVzaXNOZXdTMjAyNDAxMDkSCUQwMjI5ODUzNRoIOXd3anl4dzM=Availablefrom北京万方数据股份有限公司.

[42] Wentao W. (2018). Reflection on the prohibition of "foreign garbage" under the perspective of environmental justice. Inner Mongolia Environmental Sciences.

[43] Shuwen W., Jiali W., Hui W. (2016). Influence of foreign garbage to ecological environment in China and analysis on the risk management and control of Customs. China Population,Resources and Environment.

[44] Zi-yan H. (2020). Changes in the import system of solid waste: necessity, challenges and its countermeasures. J. North China Electr. Power Univ. (Soc. Sci.).

[45] Jianguo L. (2018). Garbage classification and prohibition of ' foreign garbage ' entry. China Policy Review.

[46] Qianwen S. (2019). China 's ban on ' foreign waste ' promotes a new pattern of global garbage trade. Finance Econ..

[47] Jing L. (2021). China 's total ban on the entry of solid waste. Ecol. Econ..

[48] Haoyu X., Wei Y., Junshi W., Ruyue Z. (2022). Research on impact of China's ban on the import of foreign garbage on global solid waste treatment. Industrial Safety and Environmental Protection.

[49] Heyang W. (2018). The global impact of China 's ' foreign waste ' ban. Ecol. Econ..

[50] Bin Z., Liping L., Li Z. (2018). Research on the economic impact of the ban on imported solid wastes entry—taking unsorted waste paper as an example. Environ. Protect..

[51] Sun N., Tabata T. (2021). Environmental impact assessment of China's waste import ban policies: an empirical analysis of waste plastics importation from Japan. J. Clean. Prod..

[52] Awan U., Nauman S., Sroufe R. (2021). Exploring the effect of buyer engagement on green product innovation: empirical evidence from manufacturers. Bus. Strat. Environ..

[53] Awan U., Sroufe R., Kraslawski A. (2019). Creativity enables sustainable development: supplier engagement as a boundary condition for the positive effect on green innovation. J. Clean. Prod..

[54] Abbasi K.R., Hussain K., Haddad A.M., Salman A., Ozturk I. (2022). The role of financial development and technological innovation towards sustainable development in Pakistan: fresh insights from consumption and territory-based emissions. Technol. Forecast. Soc. Change.

[55] Wang J., Liu Y., Wang W., Wu H. (2023). How does digital transformation drive green total factor productivity? Evidence from Chinese listed enterprises. J. Clean. Prod..

[56] Ren W. (2021). Research on dynamic comprehensive evaluation of allocation efficiency of green science and technology resources in China's marine industry. Mar. Pol..

[57] Cai Q., Chen Y., Lin K. (2020). Does access to credit availability encourage corporate innovation?—evidence from the geographic Network of banks in China. Econ. Res. J..

[58] Qian X., Liu Z., Chen Q. (2021). The impact of multi-level market demand on the innovation of manufacturing enterprises. Econ. Perspect..

[59] Yuan B., Li C. (2018). Innovation-driven Chinese industrial green total factor productivity under environmental regulation. Ind. Econ. Res..

[60] He L., Qi X. (2022).

[61] Yu J., Zhang R., Gong X. (2022). How does digital finance affect green total factor productivity: characteristics of dynamics, identification of mechanisms and spatial effects. Mod. Econ. Sci..

[62] Chao-feng L., Xiao Y. (2020). Can environmental regulation influence the location choice of FDI in China?-a quasi-natural experiment of the adjustment policy of SO 2 sewage charge collection standard. China Population Resources & Environment.

[63] Yin L., Meng X., Wu C. (2022). The impact of environmental regulation on the green total factor productivity of manufacturing in the Yangtze River economy. Reform.

[64] Donghua Y., Yuting Y. (2022). Environmental Regulation,Technology innovation and green total factor productivity of manufacturing. Journal of Eco-Civilization Studies.

[65] Zhang F., Shi Z., Wu G. (2022). The impact of digital economy and environmental regulation on green total factor productivity. Nanjing J. Soc. Sci.

[66] Fang G., Shen K., Li X., Shi J. (2021). Supply strategy of fiber sources for China's paper industry under policies of restriction us⁃ age of plastic and banning solid wastes importation. China Pulp Pap..

[67] Cheng H. (2020). The impact of China 's new policy on solid waste import on waste paper trade - based on historical data analysis. Journal of Hubei University of Economics(Humanities and Social Sciences).

